# Alport Syndrome and Oral Mucous Membrane Pemphigoid: An Interesting Case

**DOI:** 10.7759/cureus.41519

**Published:** 2023-07-07

**Authors:** Vasileios Zisis, Dimitrios Andreadis, Eustratios Kasimatis, Eustratios Vakirlis, Athanasios Poulopoulos

**Affiliations:** 1 Oral Medicine/ Pathology, Aristotle University of Thessaloniki, Thessaloniki, GRC; 2 Oral Medicine/Pathology, Aristotle University of Thessaloniki, Thessaloniki, GRC; 3 Nephrology, Ippokrateio General Hospital of Thessaloniki, Thessaloniki, GRC; 4 Dermatology, Ippokrateio General Hospital of Thessaloniki, Thessaloniki, GRC

**Keywords:** immuno suppressant, end stage kidney disease (eskd), drug induced bullous pemphigoid, mucous membrane pemphigoid, alport syndrome

## Abstract

Alport syndrome (AS) is a hereditary progressive glomerular disease associated with sensorineural hearing loss and ocular abnormalities. It is attributed to the altered structure and the subsequent dysfunction of the glomerular basement membrane (GBM) due to the mutated type IV collagen a3/a4/a5 chains. It may emerge either as an X-linked disease, the most common, or as an autosomal disease, both recessive and dominant. A female patient, 26 years old, came in 2023 to the Department of Oral Medicine/ Pathology, Dental School, Aristotle University of Thessaloniki, complaining about pain and a burning sensation in her right cheek. Her medical history revealed the diagnosis of Alport syndrome in 2016 and kidney transplantation in 2022 with extensive post-transplantation drug administration. The clinical examination revealed an ulcer, partially covered by a pseudomembrane, on the oral mucosa of the right cheek, surrounded by an erythematous border. A biopsy was taken, and the histopathological examination showed the oral manifestation of mucous membrane pemphigoid. After communicating with the attending nephrologist, the prescription of methylprednisolone was decided, and the lesions receded. The differential diagnosis included both AS-induced pemphigoid and drug-induced pemphigoid. The thorough medical history, detailed clinical investigation, lesion biopsy, and collaboration of different dental and medical specialties constitute necessary prerequisites for a successful treatment, even in immunosuppressed patients.

## Introduction

Alport syndrome (AS) is a hereditary progressive glomerular disease associated with sensorineural hearing loss and ocular abnormalities [1]. It is attributed to the altered structure and the subsequent dysfunction of the glomerular basement membrane (GBM) due to the mutated type IV collagen a3/a4/a5 chains. Type IV collagen is crucial for the stability and proper function of the glomerular basement membrane and constitutes the majority of the GBM's total protein mass. The type IV collagen chains include 6 subtypes (a1-a6) and unite into three specific heterotrimers (a1a1a2, a3a4a5, and a5a5a6) [2]. In Alport syndrome, the mutated genes COL4A3 and COL4A4 (chromosome 2) and COL4A5 (X chromosome) are responsible for creating altered type IV collagen a3, a4, or a5 chains leading in altered GBM [3,4]. The prevalence of AS ranges from one in 5,000 to one in 53,000 patients with kidney disease [5]. 70-80% of the patients exhibit X-linked Alport syndrome (XLAS) due to the altered COL4A5 gene, whereas 5% of the patients have autosomal recessive Alport syndrome (ARAS) due to the altered COL4A3 and COL4A4 genes. However, the real prevalence of AS may be much higher since type IV collagen mutations may be responsible for up to one-third of inherited kidney diseases [6]. On the histological level, the typical lesion is described as an irregularly thinning and thickening glomerular basement membrane with a lamellated appearance and a distinctive "basket-weave" pattern [7]. When the kidney biopsy showed a thin GBM, the definitions of benign familial hematuria and thin basement membrane nephropathy were applied [5]. However, these terms may be inaccurate as progressive kidney disease may be noticed after the biopsy [5]. Hence, thin GBM isn't considered a separate histological entity, and without additional signs, it hasn't any prognostic value.

Due to the range of different gene mutations, kidney disease progresses variably. The partial or complete dysfunction of the collagen chains is correlated to end-stage kidney disease (ESKD) by the age of 30 (which is also influenced by individual risk factors such as high blood pressure, high sodium diet, obesity, and smoking) [3,8,9]. The alport syndrome includes a wide range of clinical signs and appearances. Male patients with X-linked Alport syndrome and both male and female patients with autosomal recessive Alport syndrome manifest the typical clinical appearance, which includes progressive kidney disease, hematuria, proteinuria, sensorineural deafness, ocular abnormalities and aortic aneurysms, which are considered as secondary typical signs of the disease, especially in male patients with X-linked Alport syndrome [10]. In male XLAS cases, ESKD develops in 50% of the patients by the age of 25 and 90% of the patients by the age of 40. In female XLAS cases, the clinical appearance varies due to X chromosome inactivation [5]. 12% by age 40, 30% by age 60, and 40% by age 80 develop ESKD [9]. ARAS cases, regardless of sex, develop ESKD by the age of 40 [11]. In the minority of patients with autosomal dominant disease, ESKD develops in 20% of the cases when proteinuria, GBM thickening, and sensorineural hearing loss are present and in less than 1% when not [3]. AS patients with ESKD are usually excellent candidates for kidney transplantation due to their young age and lack of comorbidities [5]. Preventive kidney transplantation provides even better results, if possible since the adverse effects of kidney malfunction and hemodialysis are avoided. The 5-year survival rates are better compared to ESKD patients due to other causes. In any case, heterozygous COL4A3 and COL4A4 close relatives must not be considered donors since the donors may manifest worsening kidney function with concurrent proteinuria [4,12-14]. Also, post-transplant anti-GBM nephritis in AS patients may be an unusual yet lethal complication [15]. Mucous membrane pemphigoid (MMP) is a chronic, immunologically mediated bullous disorder affecting the skin and mucosal surfaces, including the oral cavity [16]. MMP is characterized by subepithelial blister formation, which is caused by the binding of autoantibodies to antigens found either on the keratinocyte cell surface or the basement membrane zone (BMZ). The oral mucosa is commonly affected and may precede any other involvement, while the ocular mucosa is the second most common site of involvement and may be considered a prognostic severity indicator [17].

Oral mucosa, particularly buccal mucosa, soft palate, and lips, are the first to be affected in 90% of MMP patients, whereas skin lesions are uncommon in MMP and arise only after mucosal manifestation [18]. There have been no reports on the appearance of mucous membrane pemphigoid in AS patients, and an interesting case of bullous pemphigoid in an XLAS male patient was reported, with concurrent hearing loss and kidney dysfunction [1]. This case report aims to present an interesting case where a female AS patient exhibited mucous membrane pemphigoid after ESKD and kidney transplants. 

## Case presentation

A female patient, 26 years old, came on 03/02/2023 to the Department of Oral Medicine/ Pathology, Dental School, Aristotle University of Thessaloniki, complaining about pain and a burning sensation in her right cheek. A thorough clinical examination was performed. The patient was informed consent when she was examined, giving written agreement. This form was approved by the School of Dentistry, Aristotle University of Thessaloniki, Greece, and was in accordance with Helsinki Declaration for research and patient ethics. Her medical history revealed the diagnosis of Alport syndrome in 2016, which led to ESKD and kidney transplantation on 30/06/2022. Sensorineural hearing loss and ocular abnormalities were not reported. The pre-transplantation drug administration included sucroferric oxyhydroxide, folic acid, and erythropoietin. The post-transplantation drug administration included a variety of medications aiming mainly to suppress the immune system, adjust the blood pressure and prevent infections: methylprednisolone 16mg twice per day, tacrolimus 4mg twice per day, mycophenolic acid 500mg twice per day for first seven months after the kidney transplantation and 250mg twice per day afterward, carvedilol 12.5mg twice per day, omeprazole 20mg twice per day, valganciclovir 900mg once per day, alfacalcidol 0.5mg once per day and a combination of trimethoprim/sulfamethoxazole 960mg every Monday, Wednesday and Friday.

The clinical examination revealed bilateral ulcers, partially covered by a pseudomembrane, on the oral mucosa of both cheeks, surrounded by an erythematous border. The Nikolsky sign was positive because when applying pressure with the finger, with a 450-degree angle, on the mucosa adjacent to the lesion, a blister was formed due to disruption of the intercellular adhesion. Subsequently, a biopsy was taken from the ulcer on the oral mucosa of the right cheek, from the peripheral area to include the transition from the ulcer to the nearby, clinically normal-appearing mucosa. The histopathological examination showed subepithelial clefts with an infiltrate of lymphocytes, typical of mucous membrane pemphigoid. Based on the level of blistering, autoimmune bullous diseases may be subdivided into two groups: pemphigoid diseases, characterized by subepithelial blistering, and pemphigus diseases, characterized by intraepithelial blistering. The differential diagnosis of subepithelial blistering diseases includes mainly the mucous membrane pemphigoid, the bullous lichen planus, and chronic ulcerative stomatitis. After communicating with the attending nephrologist, higher doses of methylprednisolone were prescribed. The patient received 48mgr daily, 32mg at 09.00 am and 16mg at 15.30 for 10 days. Afterward, the dose was gradually tapered by 4mg every 5 days, reaching the initial post-transplantation dosage of 32mg methylprednisolone daily (Table 1). 

**Table 1:** Therapeutic regime for MMP for the first 10 days and the subsequent adaptation every five days. MMP: Mucous membrane pemphigoid

09.00 a.m.	15.30 p.m.
32mg	16mg
28mg	16mg
24mg	16mg
20mg	16mg
16mg	16mg

**Figure 1:**
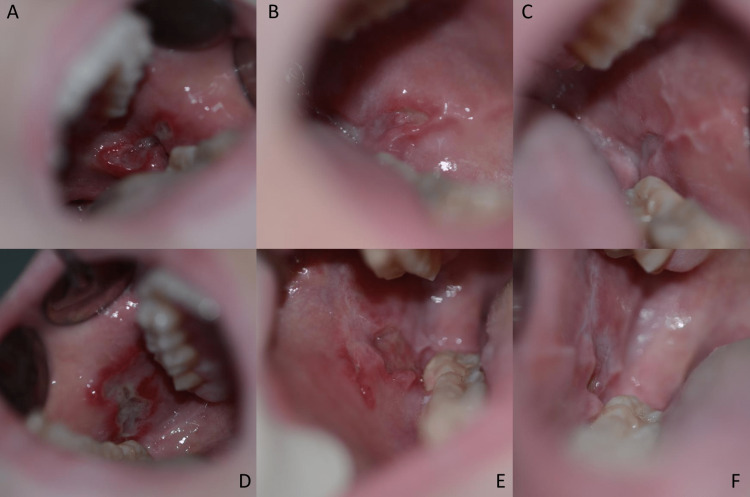
Clinical images from the bilateral lesions on the buccal mucosa. A: left buccal mucosa/ initial condition, B: left buccal mucosa/ after 3 weeks, C: left buccal mucosa/ after 6 weeks, D: right buccal mucosa/ initial condition, E: right buccal mucosa/ after 3 weeks, F: right buccal mucosa/ after 6 weeks.

The patient had weekly checkups in the university clinic to observe her clinical situation and adjust, if necessary, the therapeutic regimen. She responded to the treatment, and the oral lesions receded (Figure 1).

## Discussion

The literature shows a case report of bullous pemphigoid in an XLAS male patient [1]. The main similarity between this case and ours is the kidney disease due to AS, whereas the differences include the sex, age, and ethnicity of the patient, the type of pemphigoid disease (a 48 years old Japanese male with bullous pemphigoid versus a 26 years old greek female with mucous membrane pemphigoid) as well as the fact that our female patient eventually required kidney transplantation, The differential diagnosis mainly included lichen planus, pemphigus diseases and pemphigoid diseases including the drug-induced pemphigoid mainly due to immunosuppressants (the literature involves, from the drugs prescribed to the patient, in the pathogenesis of drug-induced pemphigoid the immunosuppressive drugs everolimus and sirolimus (analogs of tacrolimus) as well as the proton pump inhibitor omeprazole) [19]. In any case, the literature either on AS-induced pemphigoid or on immunosuppressant-induced pemphigoid remains limited. Additionally, anti-a5 (type IV) autoantibodies have been linked in two studies to the formation of subepidermal blisters. IgG autoantibodies against a 185-kDa polypeptide of the noncollagenous 1 (NC1) domain in the 5 (IV) chain caused subepidermal bullous eruptions with IgG and C3 deposits along the dermal-epidermal junction of the skin [20]. In addition, subepidermal blisters with linear IgA and C3 deposits along the dermal-epidermal junction appeared due to autoantibodies against the 185- to 190-kDa antigen of the 5 and 6 chains [21]. Except for systemic lupus erythematosus (SLE), antibodies against native human type IV collagen were rarely reported [19]. In cases of rheumatoid arthritis, scleroderma, and SLE, antibodies against denatured human type IV collagen were identified [22]. Our patient exhibited MMP despite the extensive, post-transplantation immunosuppressive regimen. The treatment with methylprednisolone succeeded in alleviating the patient and provoking the recession of the lesions. Subsequently, the stable daily dosage of 32mgr will keep the patient stable, with regular bi-monthly checkups to observe the clinical situation. The limitations of this report include the lack of examination of the genetic background of the patient due to the patient's inability to cover the expenses, the inability to access the medical history of other family members, the lack of direct and indirect immunofluorescence, and the lack of ELISA for BP180 and BP230.

## Conclusions

The thorough medical history, detailed clinical investigation, lesion biopsy, and collaboration of different dental and medical specialties constitute necessary prerequisites for a successful treatment, even in immunosuppressed patients. In the case of Alport syndrome, further research is required to shed some light on its pathogenetic mechanism and detailed clinical investigations to classify the wide variety of signs and secondary clinical entities that are induced by it. Rare genetic diseases in medically compromised patients may create a complex clinical situation where it is ambiguous whether the genetic mutation caused it directly or indirectly through concurrent drug administration.
